# Palm-Print Pattern Matching Based on Features Using Rabin-Karp for Person Identification

**DOI:** 10.1155/2015/382697

**Published:** 2015-12-01

**Authors:** S. Kanchana, G. Balakrishnan

**Affiliations:** ^1^Anna University of Technology, Trichy, Tamil Nadu, India; ^2^Indra Ganesan College of Engineering, Trichy, Tamil Nadu, India

## Abstract

Palm-print based individual identification is regarded as an effectual method for identifying persons with high confidence. Palm-print with larger inner surface of hand contains many features such as principle lines, ridges, minutiae points, singular points, and textures. Feature based pattern matching has faced the challenge that the spatial positional variations occur between the training and test samples. To perform effective palm-print features matching, Rabin-Karp Palm-Print Pattern Matching (RPPM) method is proposed in this paper. With the objective of improving the accuracy of pattern matching, double hashing is employed in RPPM method. Multiple patterns of features are matched using the Aho-Corasick Multiple Feature matching procedure by locating the position of the features with finite set of bit values as an input text, improving the cumulative accuracy on hashing. Finally, a time efficient bit parallel ordering presents an efficient variation on matching the palm-print features of test and training samples with minimal time. Experiment is conducted on the factors such as pattern matching efficiency rate, time taken on multiple palm-print feature matching efficiency, and cumulative accuracy on hashing.

## 1. Introduction

Biometrics has been receiving a mushroom growth with the increase in the civilian, military, and forensic applications, which involves with the significant identification of persons on the basis of biological or behavioral characteristics. Nowadays, a plethora of biometrics-based methodologies is available, and among them palm-print identification has received greater attention. Singular Value Decomposition (SVD) [1] based minutiae matching method involved in the recognition of individuals through finger vein though resulted in improved identification of individuals with compromised accuracy. Two categories of minutiae called bifurcation point (BP) and ending point (EP) were applied from the skeletonized binary finger vein images and extracted three local descriptors, local average intensity (LAI), local intensity deviation (LID), and local extensive binary pattern (LEBP). These three features were used for efficient minutiae matching. Though the method was proven to be robust and reliable, accuracy of minutiae matching algorithm remains unaddressed.

Multimodal Sparse Representation (MSR) [2] method reduced noise and occlusion through correlations and coupling information though it handled nonlinear variations but at the cost of accuracy which is included in our method through Rabin-Karp based Palm-Print Pattern matching algorithm. MSR presented a robust feature level fusion algorithm to handle different dimensions of different modalities through their sparse coefficients. The method was also proved to be robust to occlusion and noise by introducing an optimization framework that handled nonlinearity through kernelization. Though quality measure for multimodal fusion using join sparse representation was handled in an efficient manner, the rate of accuracy remained unsolved.

One of the major problems faced in person identification based on palm-print images is the individual identification with high confidence. The significance of the problem lies in the effective feature matching using palm-print images. To address the problem related to feature (i.e., principle lines, ridges, minutiae points, singular points, and textures) matching higher accuracy, in this work, Rabin-Karp Palm-Print Pattern Matching (RPPM) method is presented. The problems related to the existing method are that though reliability of palm-print matching was robust to noise and occlusion, effective palm-print features matching was not addressed. In order to address an effective palm-print features matching Aho-Corasick Multiple Feature matching procedure is applied in the proposed RPPM method.

The contributions of our palm-print feature matching algorithm are summarized as follows. First, we learn different angle of position using double hashing with finite set of bit values as an input text and avoid the hash collision to improve the accuracy of pattern matching. The value of palm-print pattern with different features is evaluated using the double hashing, which motivates us to exploit different angle of position and use it for palm-print feature matching. Second, multiple patterns of features between the test and training samples are matched simultaneously using the Aho-Corasick Multiple Feature matching procedure. It is different from the widely used multiple feature matching, where the Aho-Corasick Multiple Feature matching procedure locates the position of the features with finite set of bit values as an input text. The finite set of bit values learnt from multiple features makes our palm-print feature matching more effective but involves lengthy search process which is double the time of the actual single palm-print feature matching. Third, with an efficient bit parallel ordering, a high level time efficient method is constructed on matching the palm-print features of test and training samples with minimal time.

This paper is organized as follows: Section 2 provides a review of the existing literature on palm-print biometric pattern matching.  Section 3 provides the details of the double hashing procedure and Rabin-Karp Palm-Print Pattern Matching algorithm. In Section 4, we present the experimental setup with description of database. In Section 5, results analysis is discussed with parametric definitions. Finally, Section 6 includes the concluding remarks.

## 2. Related Work

Latent palm-prints include certain amount of significant critical evidential measure for applications related to forensic as it is probably estimated that about 30 percent of the latents obtained through crime scenes are those of palm-prints. Minutiae propagation algorithm introduced in [3] provided measures for reducing the time taken during palm-print matching. Reconstruction algorithm designed in [4] provided an insight into different types of attacks using fingerprint recognition system. However, the features considered were limited which is solved by applying different features like principle lines, ridges, minutiae points, and textures in our method. Robust key point detection using Scale Invariant Feature Transform (SIFT) [5] resulted in improvement in retrieval performance, but at the cost of time which has been addressed in our method using Time Effective Bit Parallel Ordering. Another latent fingerprint matching introduced in [6] provided an insight into improving latent matching accuracy using feedback paradigm. However, the features considered were restricted which have been included in our work.

Segmentation and enhancement of palm-prints in [7] were designed with an objective of providing robustness and accuracy using Total Variation decomposition model, but at the cost of time. Nonmatch Probability (NMP) [8] was designed to improve the accuracy of matching being performed using fingerprint characteristics but did not consider different features. The above two said issues were addressed in our work by introducing different features and effective time model. Multispectral Palm-Print Recognition (MPR) [9] was introduced to improve the personal identification system using Minimum Distance Classifier (MDC) scheme and Weighted Majority Voting (WMV) algorithm. With the objective of improving the personal identification using left and right palm-print images, specialized algorithm was designed using score-level fusion in [10]. However, accuracy was compromised which has been introduced in our work using double hashing procedure.

Several research works for palm-print recognition has been performed with the aid of 2D palm images. Though higher amount of accuracy can be achieved using 2D palm-print recognition, much 3D palm structural information is lost. 3D palm-print classification using global features was introduced in [11] with the objective of reducing the noise. However, multiple features were not addressed which is solved through our method that applies for multiple features. Morphological operation was applied in [12] with the objective of improving the total success rate using features like principle lines, wrinkles, ridges, singular points, and minutiae points.

A hybrid biometric cryptosystem introduced in [13] solved the problems and issues related to security against different attacks using fuzzy commitment approach. However, the false positive rate increased with the increase in more sophisticated keys which is solved in our method using Aho-Corasick procedure. Centered Discretion Hashing technique designed in [14] provided insights into reducing the tolerance region with the application of Principal Orientation Pattern (POP) and Orientation Pattern (OP).

To minimize the vulnerabilities related to palm-print, biometric template security model was introduced in [15]. Descriptor Based Hough Transform (DHT) [16] included mechanisms for improving the matching accuracy using palm-print matching algorithm. However, matching performance was compromised with differing features taken into account. Palm-print classification using *k*-nearest neighbor is used in [17] addressing the issues, with the objective of reducing the noise. However, the method highly depends on threshold value which is addressed through different features using double hashing table.

More specifically, the recognition of palm-print is based on either low resolution or high resolution of images. Minutiae cylindrical code applied in [18] provided an efficient feature extraction method for palm-print recognition. Quantum algorithms introduced in [19] provided an exponential speed-up using Quantum Fourier Transform (QFT) with the objective of increasing the matching accuracy. In [20], Fractional Differential Algorithm (FDA) was applied to reduce the noise. However, accuracy was not achieved in the above-stated algorithms. To average the equal error rate in [21], Dempster-Shafer fusion theory was applied to unique features using mean curvature features.

Based on the aforementioned methods and techniques, we design an efficient pattern matching procedure for palm-print images using Rabin-Karp Palm-Print Pattern Matching algorithm.

## 3. Rabin-Karp Palm-Print Pattern Matching Based on Features

**Figure 1:**
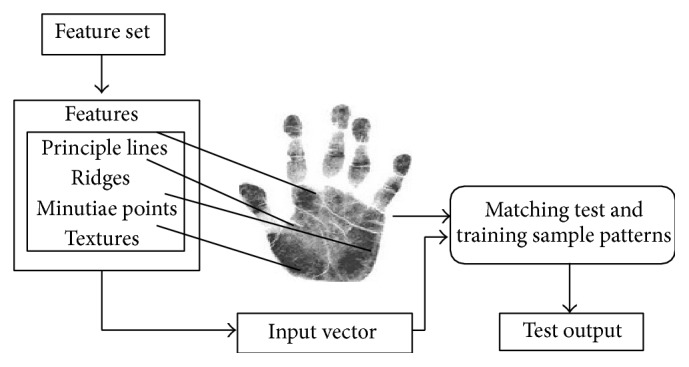
Pattern match based on features.

Biometric palm-print image pattern matching uses Rabin-Karp Palm-Print Pattern Matching (RPPM) method which is constructed with the help of double hashing method with different angle of position. RPPM method is applied for multiple features matching using Aho-Corasick procedure and time efficient feature matching using bit parallel ordering. The concept of bit pattern matching with features such as principle lines, ridges, minutiae points, and textures is performed simultaneously in RPPM method. The pattern matching with the features is clearly shown in Figure 1.

As shown in Figure 1, the pattern matching based on features for palm-print pattern uses the aggregation of features using RPPM method. Palm-print matching is carried out by making use of the features with precise characteristics to attain high accuracy rate. The feature principle line is taken as a significant part on palm-print pattern matching in our proposed method RPPM. Minutiae points are based on position, path, and direction of ridges. Texture is used to separate the feature into the region of interest and performs the matching process.

The RPPM forming several palm-print images “*I*” uses the double hashing procedure to retrieve the result with collision-free hash values on different angle of test and training sample position. The modifications in the position and direction are also easily matched by Rabin-Karp through double hashing. Multiple feature matching is done effectively using Aho-Corasick procedure. The feature bit matching of the palm-print image uses the Time Effective Bit Parallel Ordering method to perform the matching nonlinearly to reduce the time complexity.

**Figure 2:**
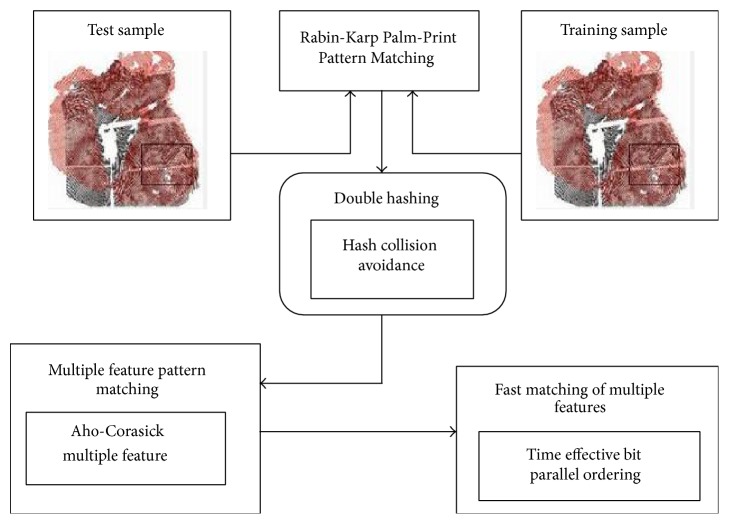
Overall structural diagram of RPPM method.

The overall structural diagram of RPPM method is shown in Figure 2.

As illustrated in Figure 2, test samples are given as input to the RPPM method. Initially, the RPPM introduces the double hashing technique to reduce the collision rate. Collision is said to occur because different angular position of minutiae points results in differing matching rate while working with the test and training sample images. So, double hashing technique is applied to RPPM method with the objective of reducing the collision rate.

Subsequently, feature based pattern matching is carried out using the Aho-Corasick Multiple Feature. The objective behind the application of Aho-Corasick Multiple Feature is to present multiple features while performing pattern matching. To perform multiple pattern matching, Aho-Corasick Multiple Feature is applied to the RPPM method. With the search being linear using Aho-Corasick Multiple Feature, resulting in time complexity, Time Effective Bit Parallel Ordering method is employed for the fast matching of the palm-print features.

### 3.1. Double Hashing Procedure

The goal of the RPPM method is to identify the consistent and diverse features in palm-print by performing the double hashing procedure. Double hashing procedure in RPPM resolves the hash collisions with different values in hash table. The double hashing for RPPM method is formularized as

(1) Double  Hashing=k1h1i,j+k2h2i,jmod⁡T.

In (1), two hash functions *h*_1_ and *h*_2_ are used widely for efficient identification of the matched features with “*i*” and “*j*” representing the pixels of palm-print images that are tested. To fetch the accurate results without collisions, “*k*_1_” and “*k*_2_” is the alternate keys (i.e., based on the positional change) used with the aid of “*T*” that represents the hash table for palm-print feature matching.

If the palm-print features are not matched with the single hash value, then the other hashing is carried out with different hash key.

As a result, “*i*” and “*j*” probe pixels of palm-print image are analyzed through the double hash key values in Rabin-Karp Palm-Print Method. Rabin-Karp Palm-Print uses the double hash function and is represented in Table 1.

The double hashing table produces the sample form of hash key used for the palm-print feature matching. The hash keys “*h*_1_” and “*h*_2_” are placed for removing the collision resolution on the palm-print biometric individual identification. The sequence of bits for different series of palm-print image features is generated and placed in the table to perform the matching. The training samples used in the table are probe sequences to test the palm-print image features.

### 3.2. Multiple Feature Pattern Matching

**Figure 3:**
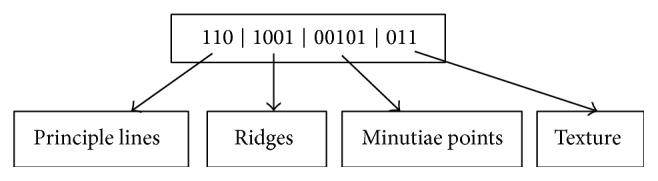
Probe sequences of bits.

Once the relevant features are identified using double hashing procedure, the next step is to perform the efficient pattern matching for multiple features. Let us assume a palm-print image of length “*n*,” with the different feature pattern “*f*” producing the best case of result on matching multiple features simultaneously. The probe sequence of bits for different image performs different separation to match the specific features. The different set of bits helps to recognize (i.e., to match), to test and train samples, and to produce more accurate biometric results for individuals. The probe sequence of bits on the double hashing table is shown in Figure 3.

Figure 3 shows the probe sequences of bits. The above double hashing based probe sequence helps to fetch multiple feature result accurately using RPPM method.

#### 3.2.1. Aho-Corasick Multiple Feature

Aho-Corasick procedure automates the transition of pattern matching of features without any backtracking process. Aho-Corasick is constructed with the double hashing table using RPPM method. This procedure finds the right function “*f*_*i*_” to match the palm-print feature pattern accurately. Each row in the double hash table identifies the hash key values “1” and “2” for that specific probe sequence of bits whereas the column path indicates the sequence of key value “1” and “2” and probe sequence bit, respectively. Followed by this, the matching process is formulized as follows:

(2) Feature  MatchingFM=f1k1,k2,f2k1,k2,…,fnk1,k2.

The features uses the Aho-Corasick data structure for matching multiple features with the hash key values. In this way multiple feature matching operation is carried out with explicit value in RPPM method.

### 3.3. Fast Matching of Multiple Features

In Aho-Corasick, the linear form of multiple features is carried out in RPPM method, but the time complexity arises on matching the palm-print features in a linear fashion. To reduce the time complexity in our proposed work, Time Effective Bit Parallel Ordering method is designed.

#### 3.3.1. Time Effective Bit Parallel Ordering

RPPM method easily constructs a nonlinear automaton to improve the matching of the test and training sample image bit in a parallel fashion. Bit parallel ordering technique in RPPM method is favorable to cut down the time taken on matching the features. Bit parallel ordering of the probe in RPPM method produces the suitable bisection for matching different combination of features in an effective manner and is formulized as

(3) $$\begin{array}{c} \text{BPO}=\min\text{tim}\left[\;{FM}_{n}\;\right]⟶\text{bit ordering}. \end{array}$$

Bit Parallel Ordering “BPO” of features for matching nonlinearity mainly depends on the ordering format. The “min⁡tim” denotes the minimum time taken on matching multiple palm-print features simultaneously. The ordering of the bit palm-print image feature is formulized as

(4) ∅bitI=δtim→nIi,jfmatch=1δtim→n+1Ii,jf−1match,else  otherwise.

The time factor on ordering the bit before performing the palm-print feature matching is provided in (4). Here, the palm-print image is represented in with “*I*” and the pixels are denoted as “*i*” and “*j*” in order to perform feature matching. The features are matched with the ordered bits of varying size of “*n*” images. The length varied features which are not matched are removed, to perform the accurate matching with the double hashing table.

## 4. Experimental Evaluation

**Figure 4:**
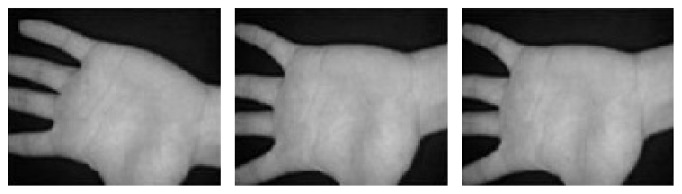
Sample palm-prints in CASIA database.

Rabin-Karp Palm-Print Pattern Matching (RPPM) method uses MATLAB coding to perform palm-print matching. Initially, the features of the palm-print with the positional angles are mentioned for effective processing. CASIA database consists of 5.502 palm-print images of both left and right palms with 8-bit gray level JPEG files confined from 312 users as depicted in Figure 4.

The pattern matching efficiency rate, time taken on multiple palm-print feature matching efficiency, cumulative accuracy on hashing, and false positive rate on matching the patterns are the factors used for experimenting evaluation. The proposed method is compared against the existing methods such as Singular Value Decomposition (SVD) [1] based minutiae matching method and Multimodal Sparse Representation (MSR) [2] method.

The pattern matching efficiency rate in RPPM is the amount of patterns efficiency matched using the double hashing procedure. The pattern efficiency rate is measured in terms of percentage and is the ratio of difference between the number of features matched and features provided as input to the total number of features provided. Consider

(5) $$\begin{array}{c} \text{PME}=\overset{n}{\underset{i=1}{\sum }}\frac{\left(f_{matched}-f_{i}\right)}{n}. \end{array}$$

From (5), the pattern matching efficiency rate “PME” is performed by the ratio of difference between the number of features matched “*f*_matched_” and features provided as input “*f*_*i*_,” where “*n*” denotes the total features provided as input. The higher pattern matching efficiency proves the efficacy of the method. The time taken on multiple palm-print feature matching is the amount of time taken to perform the feature matching for multiple palm-print features using the bit ordering. It is measured in terms of milliseconds (ms). Consider

(6) Time  for  pattern  matching=Time∅bitI.

The cumulative accuracy on hashing using RPPM method is the difference between the measured features to the actual features. Consider

(7) $$\begin{array}{c} \text{CAH}=\overset{n}{\underset{i=1}{\sum }}\left(\;{Measured}_{f_{i}}-{Actual}_{f_{i}}\;\right). \end{array}$$

The false positive rate on matching the patterns refers to the measure of good features falsely identified as bad features. It is measured in terms of percentage (%). Consider

(8) $$\begin{array}{c} \text{FPR}=\frac{\left({Good}_{f}-{Bad}_{f}\right)}{{Good}_{f}}. \end{array}$$

The false positive on matching the patterns is the ratio of difference between the good features and bad features to good features. Low false positive rate on matching the patterns confers the efficiency of the method.

## 5. Results Analysis of RPPM

The Rabin-Karp Palm-Print Pattern Matching (RPPM) method is compared against the existing Singular Value Decomposition (SVD) [1] based minutiae matching method and Multimodal Sparse Representation (MSR) [2] method. The experimental results using MATLAB are analyzed and displayed with the aid of tables and figures given below.

### 5.1. Scenario 1: Pattern Matching Efficiency

Table 2 shows the pattern matching efficiency over 21 different images provided as input using MATLAB. The changes in the pattern matching efficiency are also being observed, even in case of dissimilar images. However, the pattern matching efficiency in an increasing stage till 9 images was considered. But with an increase in the number of images to 12, the pattern matching efficiency decreased and then increased to 15 images. This is because of the different images gathered from both the male and female. As these images are not similar, the changes in the pattern matching efficiency are also being observed.

**Figure 5:**
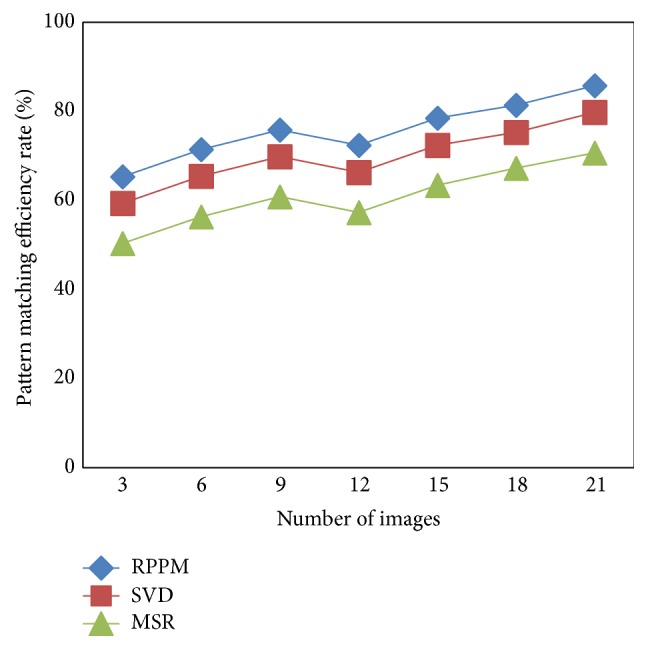
Impact of pattern matching efficiency.

Comparatively, from Figure 5, the pattern matching efficiency is improved using the proposed method RPPM with the application of double hashing procedure. On working with the test and training sample images, the analysis uses different angular position of minutiae points and results in higher pattern matching efficiency rate by 7–9% compared to SVD [1]. In addition, using RPPM method, based on the positional changes of the features, with the aid of alter key and hash table, the features are matched not only with the single hash value but with different hash key resulting in the improvement of pattern matching efficiency by 17–23% compared to MSR [2].

### 5.2. Scenario 2: Time for Pattern Matching

**Figure 6:**
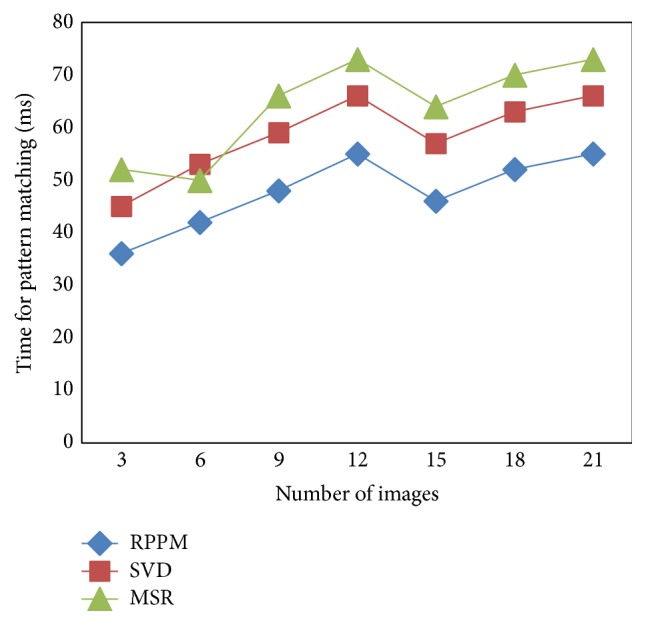
Impact of time for pattern matching.

The convergence plot for 21 images is depicted in Figure 6 and Table 3. We could observe that the proposed RPPM method achieved minimum time for pattern matching when compared to other methods. We also figure out that, in Figure 6, the proposed RPPM method shows an increase in the beginning of the convergence graphs with the setting of images with updated training and test database during the early iterations. However, when the number of images was 15, the time for pattern matching reduced in a drastic manner because of the Bit Parallel Ordering method.

Figure 6 shows that the time for pattern matching increases with the increase in the number of images and shows that a drift decrease occurs when 15 images were used. The time taken on multiple palm-print feature matching efficiency is reduced with the application of Time Effective Bit Parallel Ordering method.

The Time Effective Bit Parallel Ordering method in RPPM effectively constructs a nonlinear automaton in a parallel manner for the test and training sample images by producing suitable bisection and therefore reducing the time taken on multiple palm-print feature matching by 20–26% compared to SVD [1].

Moreover, the length varied features of test and training sample images that are not matched are removed using RPPM and as a result the time taken is reduced on multiple palm-print feature matching by 19–44% compared to MSR [2].

### 5.3. Scenario 3: Cumulative Accuracy on Hashing

The Rabin-Karp Palm-Print Pattern Matching (RPPM) method is compared with the two existing methods in terms of cumulative accuracy on hashing in this section and is depicted in Table 4 with differing samples. The number of users ranges from 5 to 35 where the experiments were conducted using MATLAB. We can notice that the proposed RPPM method had better cumulative accuracy on hashing compared to the state-of-the-art works, respectively.

**Figure 7:**
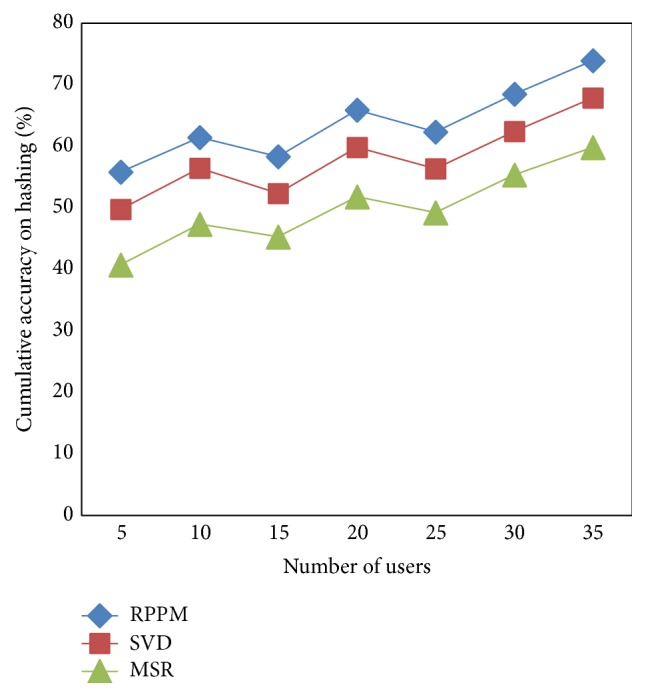
Impact of cumulative accuracy.

From Figure 7, we can notice that the RPPM method converge high accuracy on hashing than SVD [1] and MSR [2] which increases the performance measure. The cumulative accuracy on hashing is improved with the application of multiple feature pattern matching. This is effectively carried out using probing sequence of bits for different images where efficiency performs different separation to match the specific features using double hashing table. This results in the increase of cumulative accuracy on hashing using RPPM method by 8–10% compared to SVD and 19–27% compared to MSR, respectively.

### 5.4. Scenario 4: Impact of False Positive Rate

Convergence characteristics of measure of false positive rate for 35 test images with varying principle lines, ridges, minutiae points, and textures are considered and compared with two other methods and are shown in Table 5.

**Figure 8:**
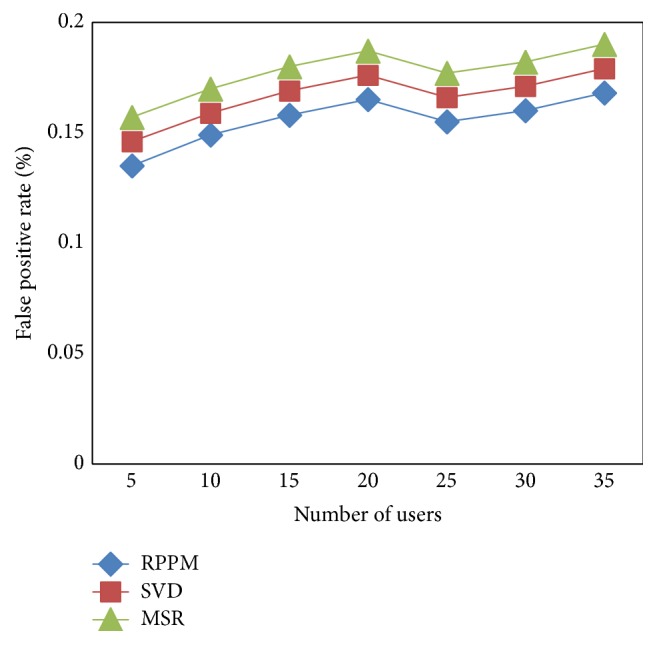
Impact of false positive rate.

The targeting results of false positive rate on matching the patterns using RPPM method are compared with two state-of-the-art methods [1, 2]. In Figure 8, visual comparison is presented based on the initialization of features. Our method differs from the SVD [1] and MSR [2]. We have incorporated competent procedure called the Aho-Corasick procedure. The Aho-Corasick procedure designs the finite state machine in an accurate manner for performing easy matching functions for multiple features without any backtracking process and therefore minimizes the false positive rate on pattern matching using RPPM method by 6–8% compared to SVD. Furthermore, the linear form of multiple feature matching operation further enhances the accuracy and therefore reduces the false positive rate on pattern matching by 13–16% compared to MSR.

## 6. Conclusion

The conventional palm-print based person identification usually designed for providing high quality pattern matching using differing features like principle lines, ridges, minutiae points, singular points, and textures with high confidence may not give satisfactory result for accuracy during pattern matching. To improve the accuracy of palm-print features matching and reduce the time taken on multiple palm-print features matching efficiency on palm-print images, Rabin-Karp Palm-Print Pattern Matching (RPPM) method based on Double Hashing procedure and enhancing multiple feature matching using Aho-Corasick Multiple Feature matching procedure has been implemented. The three-step model, feature identification using Double hashing procedure, multiple feature pattern matching using Aho-Corasick procedure, and fast matching of multiple features using time effective Bit Parallel Ordering method introduced in RPPM resulted in significant improvement over the state-of-the-art methods in terms of pattern matching efficiency, time on multiple palm-print feature matching efficiency, cumulative accuracy on hashing, and false positive on matching the patterns.

## Figures and Tables

**Table 1:** Double hashing table.

Hash 1 (*h*_1_) key	Hash 2 (*h*_2_) key	Probe sequence of bits for different palm-print images
Training Image		
*k*_1__1234	*k*_2__876	1101001001
*k*_1__556	*k*_2__3567	1011110101
*k*_1__5785	*k*_2__897	10101010001

**Table 2:** Tabulation for pattern matching efficiency.

Number of images	Pattern matching efficiency (%)		
	RPPM	SVD	MSR
3	65.36	59.33	50.32
6	71.43	65.4	56.39
9	75.85	69.82	60.81
12	72.35	66.32	57.31
15	78.45	72.42	63.41
18	81.33	75.3	67.29
21	85.75	79.72	70.71

**Table 3:** Tabulation for time for pattern matching.

Number of images	Time for pattern matching (ms)		
	RPPM	SVD	MSR
3	36	45	52
6	42	53	50
9	48	59	66
12	55	66	73
15	46	57	64
18	52	63	70
21	55	66	73

**Table 4:** Tabulation for cumulative accuracy on hashing.

Number of users	Cumulative accuracy on hashing (%)		
	RPPM	SVD	MSR
5	55.83	49.8	40.75
10	61.45	56.42	47.37
15	58.35	52.32	45.27
20	65.88	59.85	51.80
25	62.35	56.32	49.27
30	68.45	62.42	55.37
35	73.88	67.85	59.8

**Table 5:** Tabulation for false positive rate.

Number of users	False positive rate (%)		
	RPPM	SVD	MSR
5	0.135	0.146	0.157
10	0.149	0.159	0.170
15	0.158	0.169	0.180
20	0.165	0.176	0.187
25	0.155	0.166	0.177
30	0.160	0.171	0.182
35	0.168	0.179	0.190
